# Examining the Research on Business Information-Entropy Correlation in the Accounting Process of Organizations

**DOI:** 10.3390/e23111493

**Published:** 2021-11-11

**Authors:** Emilio Abad-Segura, Mariana-Daniela González-Zamar, Massimo Squillante

**Affiliations:** 1 Department of Economics and Business, University of Almeria, 04120 Almeria, Spain; 2 Department of Education, University of Almeria, 04120 Almeria, Spain; 3 Department of Law, Economics, Management and Quantitative Methods, University of Sannio, 82100 Benevento, Italy; squillan@unisannio.it

**Keywords:** entropy, business information, organization, accounting, scientific production

## Abstract

Open business organizations, where information flows, is shared, and exchanged, are more prepared to adapt and survive chaos, uncertainty, and entropy, so they will be more predisposed to change management. The aim of this study is to analyze research trends at the international level on business information–entropy correlation in the accounting process of organizations. Mathematical and statistical techniques were applied to 980 articles during the period 1974–2020, obtaining results on the scientific productivity of the driving agents of this topic: authors, research institutions, countries/territories, and journals. Five lines of research were identified during the period analyzed, which mainly study information theory, maximum entropy, information entropy, decision-making, and enthalpy. Future research should focus on analyzing the evolution of this topic, which forms new thematic axes related to bitcoin market efficiency, business hierarchy information, business model evaluation systems, catastrophic economic collapse, corporate diversification, CSR reports affecting accounting conservatism, economic income accounting, and information loss. Currently, the research presents an upward trend, which allows a growing interest in the subject to be deduced in the academic and scientific community worldwide.

## 1. Introduction

Entropy acts as a physical principle on any system and environment and, among them, the business environment. The natural tendency within organizations is disorganization, loss of relationships, and increased general chaos that eventually leads to failure [1,2]. In this context, a system is the set of entities characterized by certain attributes, which have relationships with each other and are located in a certain environment, according to a certain objective [3], whereas entropy refers to a measure of the amount of restrictions that must exist in a system for a process to take place and also determines its direction [4].

In a company, entropy affects the non-formal aspects that take place in the organization, such as its culture, work climate, conflict management, decision-making, communication processes, teamwork, level of empathy, necessary skills, and error management, among others [1,5,6,7].

At present, in the face of situations of market change and uncertainty, entropy in companies is constant, although at the same time it can be an element of evolution since new business objectives arise. In this order, entropy allows us to know that there are factors that trigger chaos within companies and the tools to prevent the alteration or loss of information. On the other hand, it allows, by importing more energy from the environment, for knowledge to be learned and unlearned [8,9].

According to the second law of thermodynamics, entropy fundamentally damages isolated systems, so it is possible to distinguish between open organizations and closed organizations (isolated system). Isolated systems tend toward disorder, that is, things tend toward chaos over time. In a closed system, entropy tends to be positive, so the system does not exchange energy with its environment [1,10]. In an open system, entropy tends to be negative, which is why there is an exchange of energy between the system and its environment, in which the resources used are taken from the external environment. This indicates that the more closed an organization is, that is, the less relationship it has with its environment, the more it will be condemned to an increase in entropy, whereas the more open it is, the more capacity it will develop to adapt to changing circumstances, seeking balance [2,11].

In this sense, the systems approach is described as (i) a design methodology: Decision makers must consider the impact of their actions thoughtfully and in advance, that is, systems must be planned, and must not be allowed to just happen; (ii) a common conceptual framework: The systems have originated in divergent fields, although they have characteristics in common; (iii) a new kind of scientific method: The medium is made of physical entities and living systems that share properties, and their respective attributes are so different that applying the same methods to both leads to misconceptions, and therefore, the method scientific must be complemented with new methods that can explain the phenomenon of living systems; (iv) organization theory: The systems approach allows organizations a new way of thinking in which an organizational systems theory has to consider the organization as a system whose operation is explained in terms of systemic concepts; (v) management by systems: When the systems approach is applied in organizations, it is also necessary to use the systems paradigm to solve their problems, which allows the organization to be directed by systems; and (vi) a method related to disciplines such as systems engineering, operations research, cost efficiency, etc. [12,13,14,15,16].

Accounting is defined as an open system, that is, it establishes an exchange with the environment that surrounds it, so they have inputs and outputs through which they constantly exchange energy and matter with it. Hence, the link established in this type of system means that they must be adaptive to the qualities of the environment on which they depend to be able to achieve their survival. This dependency makes it so that they cannot exist in isolation and must adapt through organization and learning to external changes [17,18].

Likewise, accounting is not only an open system that has inputs, performs a process, and generates outputs, but is also a conceptual, artificial, dynamic, and complex system, since it meets the characteristics of an information system and, in this case, a financial information system, in which there are principles of accounting doctrine or theory and procedures to process data that have been structured under a functional relationship that starts from the specification of the characteristics that financial information must meet [19,20,21].

The purpose of this study is to establish the relationship or dependence between the variables of accounting information and entropy within business organizations as systems that tend toward disorder in the development of their economic activity. Hence, the accounting information provides, in an environment of uncertainty, data that meet the characteristics of usefulness, relevance, clarity, comparability, integrity, prudence, timeliness, objectivity, and reliability. Consequently, this analysis will address the gaps by contributing to the political economy literature by providing answers to the following questions:

(i) How did scientific production on the subject evolve during the 1974–2020 period?
(ii) What are the main journals that have been published on this subject and how do they collaborate with each other?
(iii) What are the collaborative relationships between the main research drivers?
(iv) What are the main lines of research developed between 1974 and 2020 and future directions?

The main objective of this study is to analyze research trends at the international level, from 1974 to 2020, on business information–entropy correlation in the accounting process of organizations. To obtain correct answers to the research questions posed, a sample of 980 articles from scientific journals extracted from Elsevier’s Scopus database were analyzed.

This study implies an examination of the scientific production and the drivers that carry out this research topic during the period 1974–2020, in addition to the detection and evolution of the main thematic axes.

Analyzing the main trends in global research and the evolution of research on business information–entropy correlation in the accounting process of organizations allows the link between science and business activity to be established in relation to the management decision-making process in an environment of economic events subject to uncertainty. The extracted results provide supporting data for the development of future research and for decision-making to research institutions or financial organizations.

After this introduction, the literature review is presented in Section 2, which discusses the main basic concepts and the relationship between them. Section 3 shows the applied methodology. Section 4 displays the results obtained and their discussion in a broad sense. Finally, Section 5 presents the main conclusions of the study.

## 2. Literature Review

Section 2 is the consequence of a previous review of the literature, and its purpose is to constitute a framework in international research on business information–entropy correlation in the accounting process of organizations. Therefore, the main interrelated terms are described to conceptualize the study in the field of knowledge, consolidate the purpose of the research, and provide a conceptual framework for the interpretation of the results.

Table 1 shows the main articles selected after reviewing the literature on the research topic, focusing on the theoretical and conceptual structure of business information–entropy correlation in the accounting process of organizations. The analysis of these studies allowed both the problem and the purpose and objective of the research to be defined, in addition to the key concepts to apply the methodology described in the following Section 3. For each article, the following are indicated: (i) reference (see the References section), (ii) year of publication, (iii) title, (iv) author or authors, (v) journal where it was published, and (vi) the key terms that were identified related to the objective of the research.

The literature reviewed provides concrete definitions for the basic concepts or study variables of this research topic (entropy, accounting, business, and information). Some reflections on these terms used in the context of this study and that have been shaped in this specific field of study are included. Hence, with the firm intention of preventing different interpretations, the basic concepts that were used in the development of the study are defined below.

The spontaneous evolution of an isolated system results in an increase in entropy. For this reason, entropy is a magnitude that provides the degree of disorder or chaos of a system, so the greater the entropy, the greater the disorder, and vice versa [31]. The universe tends to distribute energy evenly to maximize the homogeneity of the system (its entropy), but not biological beings or human organizations [32,33].

Organizations and their members have to open up to change, and their maladjustment to the economic, social, technological, and cultural reality in which we are immersed causes an increase in entropy, causing disorder and chaos. In the corporate world, entropy is evident in many companies, with disorder and chaos raging throughout the organization. The only way to control entropy in an organization is to (i) want to do it, (ii) allocate the resources to do so, (iii) control and update said resources (people and means), (iv) be prepared for the changes that will surely one day occur, (v) keep organizational structures as flexible as possible, and (vi) surround yourself with competent, flexible people with the ability to constantly adapt to new challenges at all levels of the organization [2,33,34].

What thermodynamics calls disorder is not the same as what is called disorder in an organization. Entropy in thermodynamics is the random and uniform distribution of energy, the opposite of what human societies do [30].

The first law of thermodynamics indicates that the energy that makes up a company is found in each of its individuals, and it is neither created nor destroyed, but rather transformed. Talent management tries to channel it towards the objectives of the organization, being able to say that the training processes lead towards the transformation of individual and collective energies, that is, towards a more efficient state [35,36]. Of course, it cannot be said that the energy of the employees can be irretrievably dissipated, but rather that the temperature can vary significantly as the management style changes. Although heat has been lost in the past, it can be recovered through steering action. In the first instance, it is not possible to expect to obtain more energy than was contributed, since a part will always be lost in the process. Therefore, energy needs to be promoted by another element, in this case by business management. Energy is not produced out of nowhere but arises from exchange and interaction [1,32,37].

The second law of thermodynamics refers to entropy as a concept of disorder, that is, a company can be organized through a series of organizational restrictions. The departments are organized as small, differentiated entities and in turn we can break them down into work groups [38,39]. If the management unites some departments or groups and then wants to separate them again, the question is whether everything can go back to the way it was before. Thus, when mixing boiling water and ice water in a container, it is not possible to separate them to obtain the original temperatures. In the business world it is possible due to the energy injection of the administration, although this does not mean that it is a simple task or that individuals have not been positively or negatively influenced by the experience [40,41]. When individuals of different natures are introduced into the same work group, a modification of all of them can be expected through interaction. In this case, it is necessary to study which result is useful to make the most appropriate group to achieve the planned objectives. Entropy as the unusable part of a system’s energy indicates that money does not generate energy on its own, but can be used to drive motivation and other processes necessary to produce it. The investment may be unavoidable and the money will not be recovered directly, but in the case of companies, it will return in the form of future returns [10,30,42].

The third law of thermodynamics reflects that no individual lacks energy. All workers are capable of performance, although some require more effort than others to produce the same heat. It is as if each worker has their own temperature and as if those who are colder need more time or more energy to reach the optimum degrees in a particular environment [2]. The company must consider whether the effort required compensates for the temperature obtained or whether this element can cool its surroundings, producing a loss of heat. The hottest elements inject heat into the colder ones and the colder ones act in an inverse way, something that also responds to the zero law of thermodynamics [30,43]. Without proper management, the most active elements will tend to be lost and the only way to warm up the company will be through the continuous injection of energy by management [44,45].

To define accounting as an open system, it is necessary to know (i) what a system is, (ii) what is considered an open system, and (iii) to establish why accounting is an open system. A system is a set of entities characterized by certain attributes that have relationships with each other and are in a certain environment according to a certain objective [36,46,47]. In relation to this concept, others related to it arise, so (i) an entity is what constitutes the essence of something, (ii) attributes are those that determine the properties of an entity in a qualitative or quantitative way and the relationships (structural or functional) determine the association between two or more entities or between their attributes; (iii) the environment is the set of entities, which by determining a change in their attributes or relationships can modify the system; and (iv) the objective is the projected activity, which is selected before its execution and is based on subjective assessments and technical reasoning in relation to the characteristics of the system [48,49,50].

In this context, planning at all levels of the organization is essential to control entropy in the company, both at a strategic and tactical level. Planning refers to the continuous process without interruptions by which, once the different plans have been defined and implemented, the information must be constantly updated to be able to take corrective measures if they proceed [51]. The periodic review of the set of plans will generate feedback information that we can use in subsequent planning [8].

To avoid chaos in a company, you have to know where you are going in order to plan how to do it. People, organizations, and the way they are organized have an obligation to adapt or are doomed to disappear [52]. Dogmas are history; history must be taken into account, but it is history and, in the future, what awaits us that will be different. The recipes of the past will not work in a radically new context, in a very open economy, in an increasingly globalized and competitive world, in a world where markets have assumed the authority of economic policy [53].

The idea of constant growth that prevails in traditional economic thought does not contemplate that systems tend toward chaos, toward disorder, as expressed by the second law of thermodynamics [54,55]. Conventional economics operates in a perfect system where balances are automatic and where the cost of many factors, especially energy, is zero. For this reason, the current economy is on the path of the unsustainable. Fortunately, from biophysics to ecological economics a field has begun to open that seeks to transform the vision of traditional economics.

## 3. Materials and Methods

This section describes both the bibliometric techniques applied in the study and the data inclusion and exclusion criteria to determine the sample of articles analyzed, in addition to the data processing in relation to the research objective [56].

The bibliometric methodology applies mathematical and statistical methods to the scientific literature to analyze the activity of a certain scientific field. The objective of this methodology is to search, identify, organize, and analyze the trends of a research topic [57]. During the last few decades, these techniques have encouraged the revision of scientific knowledge and, in addition, they have been used in numerous scientific fields with success [58,59,60]. The review of the scientific literature allows (i) the relationships between the articles of a research topic to be identified, (ii) the influence of the most prominent drivers to be verified, (iii) sub-disciplines to be recognized, and (iv) the evolution of an area of research interest in the scientific community to be traced [61,62].

The aim of the study is to show a vision of the dynamics of research on business information–entropy correlation in the accounting process of organizations. To do so, a quantitative analysis was carried out by applying statistical and mathematical techniques. From the main literature reviewed for the study (see Table 1), the following terms for the search string were detected: “entropy,” “accounting,” and “business.”

The Scopus database was used to apply bibliometric techniques to the sample of articles. This database was chosen because when conducting the initial search in the Web of Science (WoS) and Scopus databases, it showed a significant difference in the volume of articles in the analyzed period of 1974–2020: 205 articles were extracted from WoS and 980 articles from Scopus.

**Figure 1:**
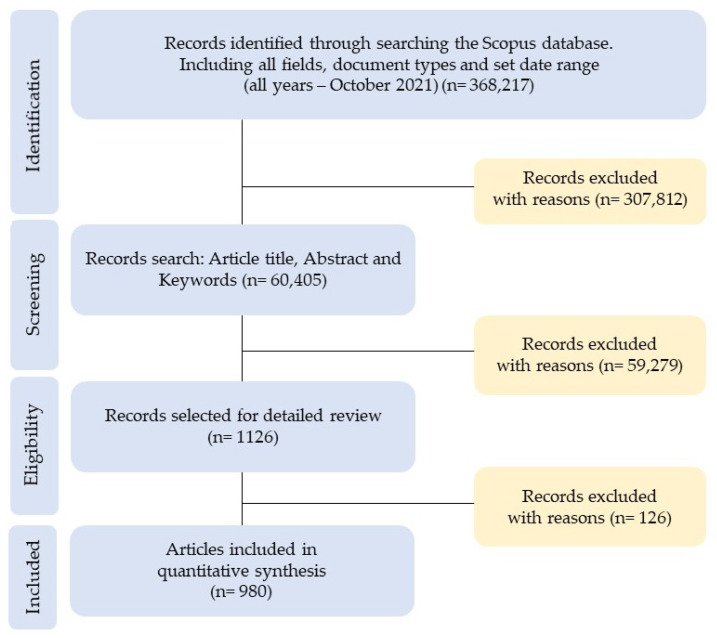
Flowchart based on the PRISMA statement.

The flowchart in Figure 1 shows the process (4 phases) followed for the selection (inclusion–exclusion) of the sample of scientific articles, according to the Preferred Reporting Items for Systematic Reviews and Meta-Analyses (PRISMA) [63].

1. Identification: 368,217 records were identified from the Scopus database, considering “all fields” for each of the key search terms (“entropy,” “accounting,” “information,” and “business”), “all types of documents,” and all data published in the “data range” (all years–October 2021).
2. Screening: the option of “article title, abstract, and keywords” was chosen in the field of each key term, and 307,812 records were excluded.
3. Eligibility: Of those 60,405 records, only “articles” were selected as the document type and “journal” as the source type, to ensure the quality of the peer review process. Furthermore, only the following subject areas were included: (i) Economics, Econometrics and Finance and (ii) Business, Management and Accounting. In this phase, 59,279 records were excluded.
4. Included: The data referring to the “all years–2020” period were selected, that is, from the first article on the research topic (1974) to the last full year (2020). In this last phase, of the 1126 records, 146 documents were excluded, so the final sample included 980 articles, both open access and non-open access.

The representation of this sample of articles is supported by the demonstrated quality of the Scopus database, with respect to the indexing protocol, and the systematic procedures of the search criteria (PRISMA).

The variables analyzed were (i) year of publication, (ii) subject area, (iii) journal, (iv) author, (v) country/territory of affiliation of the author, (vi) research institution where the author is affiliated, and (vii) the keywords that define the article. Likewise, in this study the following indicators were used: (i) activity (quantity and quality), which provides data on the volume and impact of research activities, and (ii) relationship (structural collaboration), which tracks both unions and interactions [64].

To measure the connections established between the different journals, a co-citation analysis was applied, which is used when a document cites two others, demonstrating the probability that both cited sources are related by their content. In a general sense, co-citation is a co-occurrence relationship that is established when two items from the existing literature are cited together by a third party [65].

On the other hand, to quantify the connections established between the different driving agents of this research field (authors, research institutions, and countries/territories), the co-authorship analysis was applied to evaluate the patterns of scientific collaboration, based on bibliographic data. Co-authorship analysis examines the social structure of a research field [66,67].

Likewise, to measure the connections established between the keywords that define the publications, the co-occurrence method was applied. The analysis of the keywords allowed for the detection of the main current and future research topics, since scientific articles are reduced to the set of joint appearances between the words that compose it. Co-occurrence analysis relates the proximity of two or more terms in a text unit. If two terms coexist in a sentence (they appear together in it), they are likely semantically related. In addition, the network based on the co-occurrence method offers a graphic display of the relationships of the concepts represented in the articles. This method allows the outstanding terms to be determined in a research topic and thus the main thematic axes developed to be obtained [68,69].

In this study, the VOSviewer software (version 1.6.17, Center for Science and Technology Studies, University of Leiden, Leiden, the Netherlands) was applied to the data samples to analyze the indicators of collaboration structure and relationship by co-citation, co-authorship, and co-occurrence method. These offer data on the interactions and the evaluation of the subject matters to measure the activities of the research networks [70].

The terminology used in data analysis were the following [70,71,72]:

- Item: Items are the objects of interest in the maps created, viewed, and explored with VOSviewer, which can be publications, authors, countries, institutions, journals, or keywords.
- Network: This is a set of elements together with the links between the elements.
- Cluster: This is a set of elements included in a map. These do not overlap in VOSviewer, that is, an item can belong to only one group. There may be items that do not belong to any cluster. Groups are labeled with group numbers.
- Attribute: Elements can have multiple attributes, that is, if elements have been assigned to groups, the group numbers are n attributes. Weight and score attributes stand out and are represented by numeric values.
- Weight: Weight attributes are restricted to non-negative values. Score attributes do not have this restriction. The weight of an item indicates the importance of the item. When viewing a map, items with a higher weight are displayed more prominently than items with a lower weight. Items can have multiple weights and scoring attributes. There are two weight attributes: Links and Total Link Strength.
- Links: For a given element, this indicates the number of links of an article with other articles.
- Total Link Strength: For a given element, this indicates the total strength of the links of an article with other articles.
- Occurrence: In VOSviewer, when working with keywords, the occurrences attribute indicates the number of documents in which a keyword appears.

The results obtained are useful recommendations for stakeholders involved in research on business information–entropy correlation in the accounting process of organizations who need an analysis of the scientific literature for subsequent decision-making.

## 4. Results and Discussion

### 4.1. Scientific Production (1974–2020)

#### 4.1.1. Evolution of the Number of Articles Published

Table 2 represents the evolution of scientific production at an international level during the period 1974–2020, for periods of 10 years, in the field of research of business information–entropy correlation in the accounting process of organizations.

It is observed that 66.73% (654 documents) of all the scientific production of the period was published in the last decade (2011–2020). These data indicate that it is a topic of interest to the scientific community, since the concept of entropy is understood as key in any business organization, so its control resides, among other aspects, in allocating the resources to do so, controlling and keeping these resources updated, and being prepared for the changes that are certain to occur [73].

In this same sense, it is relevant that 86.84% of the total articles published (851 of 980) were produced in the last 15 years (2001–2020), which once again confirms the growing interest of academics and researchers on this topic. During the first 27 years (1974–2000) scientific production was insignificant, since only 129 articles (13.16%) were published. These first publications were aimed at cementing the theme, with articles that dealt with accounting aggregation: user preferences and decision making [18], a thermodynamic approach to economics [30], entropy and business communication [74], and diversification strategy, profit performance, and the entropy measure [75], among others. Likewise, in the first year analyzed, 1974, only one article was published, whereas in 2020, the last year examined, 123 articles were published (12.55%).

**Figure 2:**
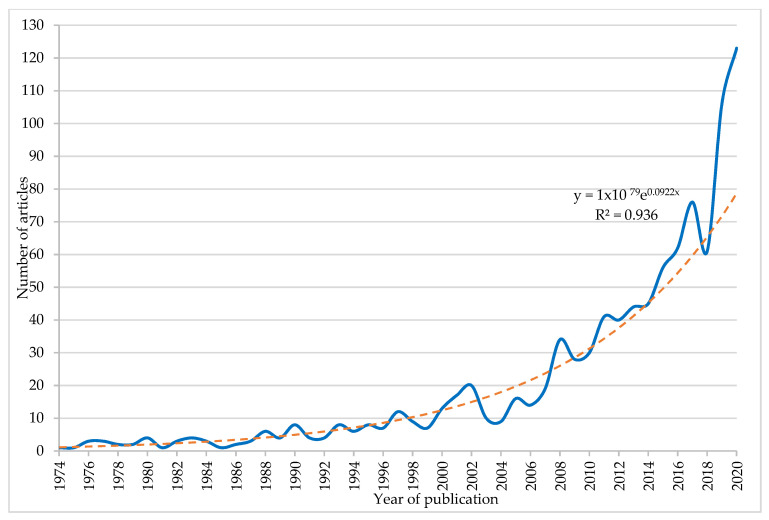
Evolution of scientific production (1974–2020).

Figure 2 shows the evolution of scientific production at a global level during the period 1974–2020 in the topic of research. The trend line was exponential and showed its goodness of fit with an R^2^ of 0.936, referring to the proportion of the variance in the dependent variable (number of articles) that is predictable from the independent variable (year of publication). Therefore, the curve represents that the number of articles on the subject of the study increased more rapidly over time.

Publication increased with the inclusion of new terms related to business planning considered an uninterrupted continuous process [76,77,78], so the information needed to be constantly updated in order to take corrective measures. Therefore, a periodic review of the set of plans generates feedback information to refine the planning [2,79,80].

The articles were published in 12 different languages. English stood out with 95.61%. It was followed with less representation by Chinese (1.33%) and Spanish (1.02%). The rest of the languages did not reach 1%. This result is related to the fact that publication in English broadens the audience globally, as was the case in most of the searches carried out in the Scopus database [81,82].

#### 4.1.2. Journals

**Figure 3:**
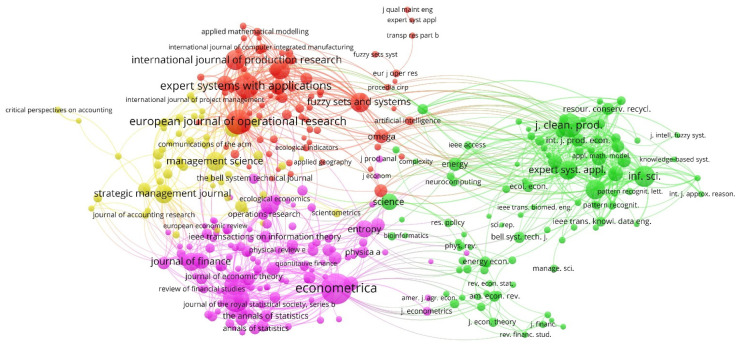
Collaboration network between journals by the co-citation method (1974–2020).

The total of 980 articles were published in 370 journals, according to the Scopus database. Figure 3 shows the network map of the journals that were published on the business information–entropy correlation in the accounting process of organizations at a global level, based on the co-citation method.

In the period analyzed (1974–2020), the journals were associated in four clusters, with a high concentration. In the visualization map, (i) the size of the circle refers to the weight of the journal, that is, the greater the weight, the larger the circle; (ii) the color of the node indicates the cluster to which the journal belongs; (iii) the lines between the elements represent links; and (iv) the distance between two nodes indicates the relationship of the journals in terms of citations links. Likewise, some journals cannot show their title on the visualization map due to the high density of the clusters and thus to avoid overlapping.

Research gradually attracted a growing number of journals during the period analyzed, as evidenced by the number of articles published and the variety of thematic areas of the interested journals on the international level.

Table 3 provides complementary information to Figure 3 of the four clusters that were detected according to the analysis of co-citations. The following is indicated for each cluster: (i) the color that represents it, (ii) the percentage of elements that comprise it over the total, and (iii) the main journal (by number of citations) and the five that stand out in each cluster, so for each one of them the weight of the links, total link strength, and citations. *Bell System Technical Journal* (Publisher: Institute of Electrical and Electronics Engineers), dedicated to the disciplines of electrical engineering, computer science, and telecommunications, stood out for its number of links (242). Regarding the total link strength, standing out with 6180 was *Journal of Cleaner Production* (Publisher: Elsevier), dedicated to environmental science. In relation to the number of citations, the journal *Econometrica* (Publisher: Wiley-Blackwell on behalf of the Econometric Society), dedicated to the discipline of economics stood out with 380.

#### 4.1.3. Main Articles Published

The first article was published in 1974, written by the author Marschak, J. (University of California, Los Angeles, CA, USA), with the title “Limited role of entropy in information economics” [83], and was published in the journal *Theory and Decision* (Publisher: Springer Nature) in the subject areas of Economics, Econometrics and Finance: General Economics, Econometrics and Finance; Social Sciences: General Social Sciences; Decision Sciences: General Decision Sciences; Arts and Humanities: Arts and Humanities (miscellaneous); Computer Science: Computer Science Applications; Psychology: Developmental and Educational Psychology; and Psychology: Applied Psychology, and had two citations.

Likewise, the most cited article (879 citations) was published in 1985 and written by the author Palepu, K. (Harvard Business School, Boston, MA, USA), with the title “Diversification strategy, profit performance and the entropy measure” [75]; it was published in the journal *Strategic Management Journal* (Publisher: Wiley-Blackwell) in the subject areas of Business, Management and Accounting: Strategy and Management, and Business, Management and Accounting: Business and International Management.

The most relevant article, which refers to the contribution that most closely matches the search terms in the Scopus database, was published in 1997, and was written by the authors Swanson, G.A. (Tennessee Technological University, Cookeville, TN, USA), Bailey, K.D., and Miller, J.G. (from University of California, Los Angeles, Los Angeles, CA, USA), with the title “Entropy, social entropy and money: A living systems theory perspective” [84], and was published in *Systems Research and Behavioral Science* (Publisher: Wiley-Blackwell) in the subject areas of Social Sciences: General Social Sciences; Decision Sciences: Information Systems and Management; and Business, Management and Accounting: Strategy and Management, and had 19 citations.

### 4.2. Analysis of the Main Drivers of the Research

#### 4.2.1. Authors

The total sample of articles was written by 2092 authors at the international level. Table 4 shows the most productive authors, that is, those with more than five articles published in the period studied. For each of them, the number of articles published on the subject of study, the research institutions with which they are affiliated, and the three most used keywords in these articles are provided. In this ranking, the eight authors affiliated with research institutions of American origin stood out, followed by four authors affiliated with institutions of European origin (the Netherlands, UK, France, and Germany). As can be seen, there were authors affiliated with more than one research institution (Golan, A.; Gossner, O.; and Tavana, M.).

The keywords most used in the works carried out by the main authors of this topic are associated with the search terms “entropy” (maximum entropy, maximum entropy in the mean, maximum entropy generalized, social entropy theory, Dirichlet process, relative entropy, computable general equilibrium (CGE), controlled stochastic process, aleatory and epistemic uncertainties), “accounting” (costs, decision-making, decision support systems, decision systems), “information” (aggregated information, incomplete information, knowledge-based systems, information theoretic methods, information theory, Kullback–Leibler information, Bayesian estimation, Bayesian learning, behavioral research, censored data), and “business” (business excellence, commerce, economic conditions, triple helix, adaptive behavior, living system).

**Figure 4:**
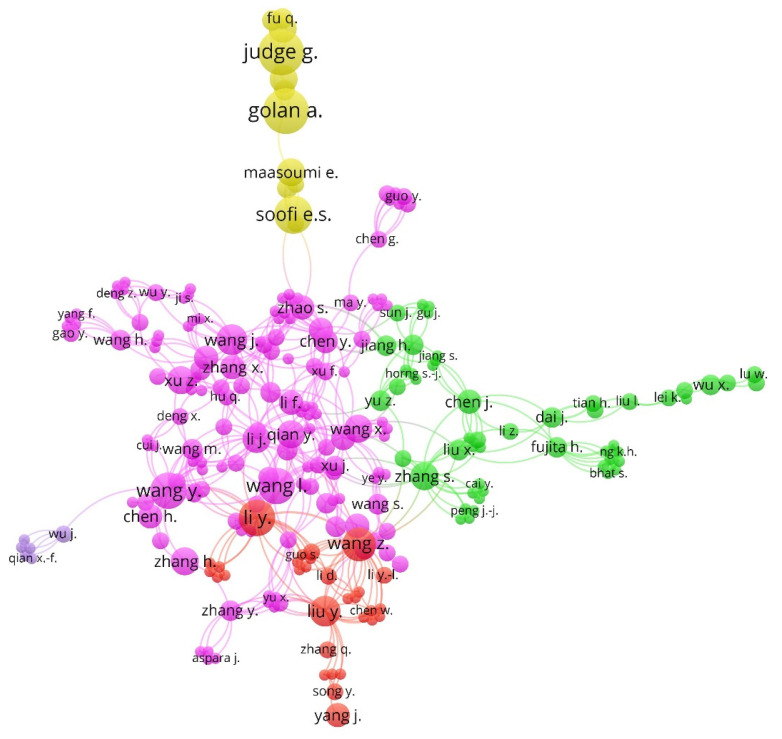
Collaboration network between authors by co-authorship method (1974–2020).

Figure 4 shows the map of cooperation between the authors based on co-authorship analyses that they published around the world on business information–entropy correlation in the accounting process of organizations. The authors were associated in five clusters. The color of each component is linked to the group of associated authors. The diameter of each circle indicates the number of articles by the author. The network showed a great dispersion in the association of authors during the period analyzed (1974–2020).

The visualization network showed a great dispersion in the association of authors by co-authorship during the period analyzed (1974–2020), where the link between authors of Chinese origin was the most noteworthy. The most central author was Wang L. (cluster 1, pink) followed by Li, Y. (cluster 3, network). The association between these authors allowed the subject to advance, among others, an integrated diagnostic framework to manage the sustainable growth of organizations [44], or the evaluation of the organizational structure of large construction companies based on the theory of entropy [85].

Table 5 shows the five clusters detected, described by color and the percentage of authors included in the total. For each cluster, the five main associated authors are presented, and for each of them the links, total strength of the link, documents, and citations are indicated, as a sample of the importance of each author within each component.

The keywords most used in the works carried out by the main research institutions of this topic are associated with the search terms “entropy” (information entropy, maximum entropy methods, transfer entropy, relative entropy, Dirichlet process), “accounting” (decision-making, decision theory, empirical analysis), “information” (Kullback–Leibler information, information theoretic methods, information theory, knowledge-based systems, Blackwell ordering), and “business” (econometrics, artificial intelligence, adaptive behavior, triple helix).

#### 4.2.2. Research Institutions

The 980 publications on the research topic were developed by authors affiliated with 1866 different international research institutions. An author can be linked to one or more than one institution.

Table 6 shows the 10 most prolific research institutions on this topic. In this ranking, four institutions of Asian origin (City University of Hong Kong, Sichuan University, Ministry of Education China, Chinese Academy of Sciences), three American institutions (University of California Berkeley, University of Wisconsin-Milwaukee, Lubar School of Business), and three European institutions (Universiteit van Amsterdam, Universidad de Oviedo, London School of Economics and Political Science) stood out.

The main keywords associated with the publications of the most relevant institutions are related to the basic concepts defined in this topic. The keywords most used in the works carried out by the main research institutions of this topic were associated with the search terms “entropy” (information entropy, maximum entropy methods, transfer entropy, relative entropy, Dirichlet process), “accounting” (decision-making, decision theory, empirical analysis), “information” (Kullback–Leibler information, information theoretic methods, information theory, knowledge-based systems, Blackwell ordering), and “business” (econometrics, artificial intelligence, adaptive behavior, triple helix). These are linked to the aspect of information provided by business activity accounting, to communication, conditional simulations, and information theory, as measures of entanglement [86,87].

#### 4.2.3. Countries/Territories

The 980 articles on this research topic were written by authors from 78 different countries/territories. The country with the most articles published on this research topic was the USA (281, 28.67%), followed by China (189, 19.29%), India (89, 9.08%), the UK (86, 8.78%), Italy (42, 4.29%), Canada (37, 3.78%), Iran (36, 3.67%), Australia (34, 3.47%), France (33, 3.37%), and Spain (32, 3.27%). The rest of the countries did not exceed 3% of the total articles published.

**Figure 5:**
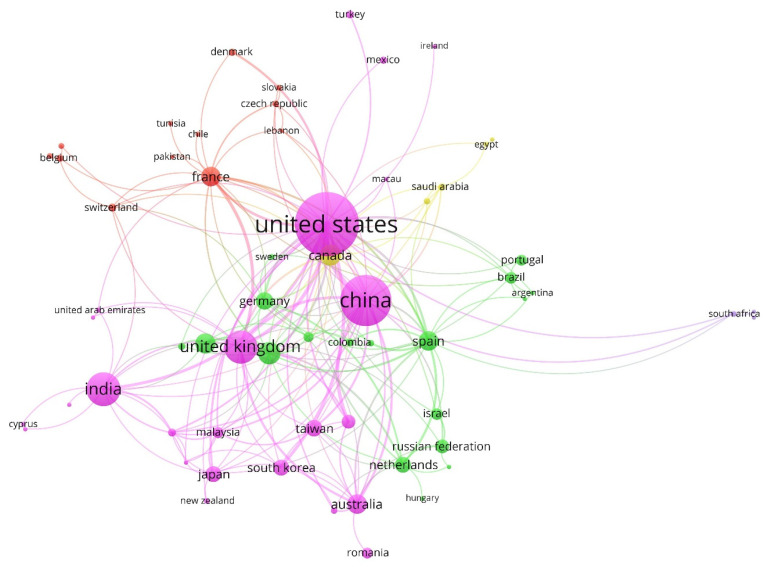
Collaboration network between countries/territories (1974–2020).

Figure 5, based on the co-authorship analysis, shows a collaboration map between the main countries/territories. The VOSviewer tool grouped them into five clusters. The different colors identify the different groups, whereas the diameter of the circle varies depending on the number of articles published by each country/territory.

Next, Table 7 shows the three most relevant countries from each of the five clusters, defined by color (see Figure 5) and the percentage of elements that compose it. Each country is defined by the number of (i) links, (ii) total link strength, (iii) documents, and (iv) citations. At a global level, the research, led by the USA and China (these countries also led cluster 1, the most central and numerous), focused on different lines of research, such as an approach to assess the quality of information accounting based on relative entropy in fuzzy linguistic environments [88], or investigating the entropy test model on the quality of internal control and accounting conservatism in financial companies in China [89].

### 4.3. Analysis of Keywords

The 980 articles selected for analysis contained a total of 5834 keywords. The 20 most prominent keywords in the sample of selected documents, according to the number of articles in which these appear, were “information theory” (85), “decision-making” (73), “maximum entropy” (56), “information entropy” (44), “fuzzy sets” (37), “Shannon entropy” (27), “mathematical models” (25), “data mining” (24), “uncertainty analysis” (23), “thermodynamics” (20), “rough set theory” (18), “uncertainty” (18), “optimization” (17), “probability distributions” (17), “forecasting” (16), “probability” (16), “efficiency” (15), “maximum entropy analysis” (15), and “economics” (14).

In this sense, an analysis of the keywords of the 980 articles selected from Scopus was carried out, based on the co-occurrence method. Five clusters were recognized that referred to different thematic axes developed during the 1974–2020 period. The analysis of the keywords through the detection of scientific communities/groups allowed the articles to be grouped into function groups of the keywords used.

**Figure 6:**
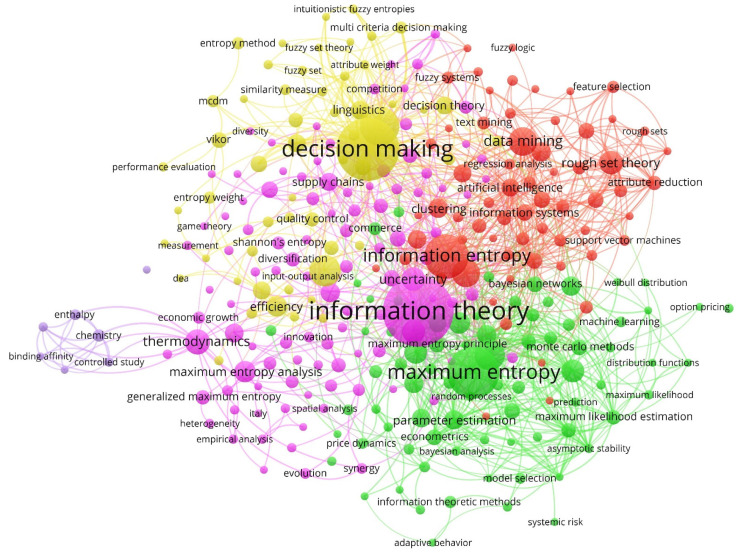
Network of keywords (1974–2020).

Figure 6 represents the network map for the keywords of the publications on business information–entropy correlation in the accounting process of organizations for the period 1974–2020, identifying each group with a color. The keywords selected in the search carried out in the Scopus database were not chosen in order not to distort the network map. Hence, the node color was applied to differentiate the groups based on the number of co-occurrences, and the size refers to the number of repetitions. The size of each cluster indicates the importance of the keywords that make up the group, and the thickness of the joining lines between two groups refers to the number of interactions established between two different communities.

Table 8 displays the five clusters that were detected based on the analysis of co-occurrences, together with the color with which each cluster is identified (see Figure 6) and the weight that each one represents of the total sample. It also includes the main keyword of each one of them, which defines the name of the cluster, and the five main keywords with which it is associated within each group. Hence, the clusters were named (i) information theory, (ii) maximum entropy, (iii) information entropy, (iv) decision-making, and (v) enthalpy. The detection of these clusters allowed for the identification of the research lines developed by the different driving agents of this topic. The interrelation of the keywords affects the idea that in open organizations, information flows are shared and exchanged so they are more prepared to adapt and survive chaos and uncertainty, that is, entropy, and its turn to change management [80,90,91].

A detailed discussion of each of the identified lines of research is provided below.

- Cluster 1: Information Theory

The first thematic axis analyzed the concept of information theory, which as a scientific principle refers to the fact that measuring the amount of information in an accounting system is complex, since accounting forms a metalanguage independent of the alphabet system (language symbols) and also independent of the language system (language) where it is circumscribed, but not independent of the entities in charge of regulating and controlling accounting, because they determine, through regulation, what must be applied with binding force, what accounting must measure and report [92,93]. Thermodynamics contributed the concept of entropy, understood as a measure of the disorder of any system; from this concept, by mathematical isomorphism, we arrive at the concept of negentropy as the opposite of entropy, that is, a measure of the order of any system, and identifying that order with the degree of organization contained in that system (negentropic theory of information) [94].

- Cluster 2: Maximum Entropy

This thematic axis developed the concept of maximum entropy, derived from thermodynamics and information theory, which describes uncertainty and disorder in a system. Self-organizing systems participate in a continuous dialogue with the environment and must adapt to changing circumstances to keep internal entropy at a manageable level [95]. The laws of thermodynamics allow the business environment to be explained in terms of team management, analyzing the fluctuation of collective energy under its principles. Numerous studies have linked business administration with concepts of energy or temperature in relation to a business organization made up of individuals of different capacities and rhythms, which ends up finding a balance after their interaction [36,79,96]. The existence of the entropy factor means that the company must constantly monitor each of the aspects to correct eventualities and avoid collapses. Entropy as a concept of disorder indicates that a company can be organized by a series of organizational constraints. Hence, the departments are organized as small differentiated entities and, in turn, can be broken down into working groups [51].

- Cluster 3: Information Entropy

The third line of research developed during the 1974–2020 period is related to the concept of information entropy. Entropy and information gain are key metrics to determine the relevance of decision-making. Hence, entropy in a company affects non-formal aspects that take place in the organization, such as its culture, work environment, conflict management, decision-making, communication process, teamwork, or the level of empathy [97,98]. The second law of thermodynamics indicates that in the processes that occur in the universe, disorder always increases, but when transferred to living beings, the entropy decreases, so that when the order of their components also decreases in entropy, it continues to increase around. That is, energy transformations and exchanges take place and, subsequently, the total entropy of the system and its environment increases.

- Cluster 4: Decision-Making

The fourth axis of knowledge developed focuses on establishing a scientific vision of decision-making in an environment of uncertainty, to build open organizations to avoid chaos and organizational failure. The elements introduced in companies, such as the economic situation, globalization, and information, are enough to generate disorder; thus, in the face of situations of market change and uncertainty, entropy in companies is constant but at the same time it is an element of evolution, since new business objectives arise [18,23,99].

- Cluster 5: Enthalpy

This fifth line of research developed the concept of enthalpy, referring to a state function of thermodynamics where the variation allows for the expression of the amount of heat put into play during an isobaric transformation in a thermodynamic system, during the transformation of which it can receive or contribute energy. In this sense, the enthalpy is numerically equal to the heat exchanged with the environment outside the system [100,101,102].

**Figure 7:**
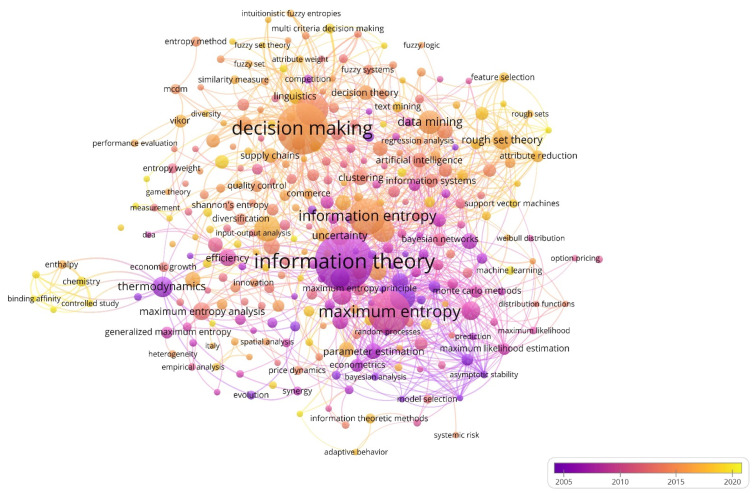
Evolution of the network of keywords (1974–2020).

Research on the subject has evolved, adapting to new international frameworks. Other concepts have been incorporated that configure different or complementary points of view and strategies and that will shape new lines of research. Figure 7 shows the evolution and full development of each group of keywords by distinguishing the period in which they were analyzed and linked to the topic. In addition, it allows us to understand the magnitude of the keywords according to the moment in which they were associated with this research topic, since the oldest provided a greater influence and are considered a reference for those that emerged later.

The sub-periods in which scientific production was developed represent a large group of keywords, which allows us to check the diversity of study lines on business information–entropy correlation in the accounting process of organizations. According to Scopus, among the most recently incorporated are the Bitcoin market efficiency, business hierarchy information, the business model evaluation system, catastrophic economic collapse, corporate diversification, CSR reports affecting accounting conservatism, economic income accounting, and information loss. The identification of these terms allows for the detection of new approaches in this research topic.

Therefore, future directions in research should focus, among others, on entropy in the exchange of information content in financial markets based on entropy analysis, on the implications of COVID-19 on the efficiency of the financial market, and on the mechanism of action of the development of the circular economy. In addition, a framework should be established to assess corporate sustainability through multi-criteria decision-making, determine the utility of entropy in estimating commercial risks, analyze barriers to the implementation of blockchain technology in supply chains, and study complex phenomena in the interdisciplinary development of accounting to capture financial records.

### 4.4. Research Implications

Regarding the academic and practical implications of the study, they can be grouped as follows:

(i) Carries out an analysis of the literature that has so far addressed the line of research on business information–entropy correlation in the accounting process of organizations.
(ii) Identifies that planning at all levels of an organization is essential to controlling entropy in the company, both at a strategic and tactical level, that is, to project the continuous process without interruptions by which, once the different plans are defined and implemented, the information must be constantly updated to be able to take corrective measures if necessary. The periodic review of the set of plans will generate information in terms of feedback that can be used in subsequent planning.
(iii) To avoid chaos in the company, you have to know where you are going, in order to plan how to do it. People, organizations, and the way they are organized have an obligation to adapt or are doomed to disappear. Dogmas are history; history must be taken into account, but it is history and in the future what awaits us will be different. The recipes of the past will not work in a radically new context, in a very open economy, in an increasingly globalized and competitive world, in a world where markets have assumed the authority of economic policy.

The notion of constant growth that dominates traditional economic thought does not contemplate that systems tend toward chaos and disorder, as expressed by the second law of thermodynamics. Conventional economics operates in a perfect system where balances are automatic and where the cost of many factors, especially energy, is zero. For this reason, the current economy leads to unsustainability, although fortunately, both biophysics and ecological economics help transform the vision of traditional economics.

## 5. Conclusions

This study analyzed the main trends in global research on business information-entropy correlation in the accounting process of organizations in the period beginning with the publication of the first article on this research topic (1974) until the last full year (2020). Statistical and mathematical techniques were applied to a sample composed of 980 scientific articles selected from the Elsevier Scopus database, applying the PRISMA systematic review protocol. This analysis made it possible to obtain the evolution of scientific production and to identify the main journals, authors, research institutions, and countries/territories that have contributed to international research on this topic.

In the analyzed period, global scientific production showed an exponential trend, since the number of articles published on the subject of study increased more quickly over time, going from one article published in 1974 to 123 in 2020. In the last decade (2011–2020), 66.73% of the documents (654) were published.

Five lines of research were identified during the period analyzed, which mainly studied information theory, maximum entropy, information entropy, decision-making, and enthalpy. Likewise, in the evolution of this topic, new thematic axes were related to the Bitcoin market efficiency, business hierarchy information, the business model evaluation system, catastrophic economic collapse, corporate diversification, CSR reports affecting accounting conservatism, economic income accounting, and information loss.

During the period analyzed, the research indicated that open companies, that is, those in which information flows are shared and exchanged, are more prepared to adapt and survive chaos, uncertainty, and the inevitable entropy, and they will be more willing to manage change. For these reasons, the main contribution of this work to the subject is that it involves an analysis of scientific production and the agents that have been stimulating research on business information–entropy correlation in the accounting process of organizations during the period 1974–2020, in addition to the identification of research directions and their transformation. The evolution in this field of study was detected from the configuration of the clusters of authors, research institutions, countries/territories, journals, and keywords, in addition to the strength of the collaborations that were developed in them. In this sense, this analysis allows for the generation of new qualitative knowledge, and stands as an entry point for future discussions.

At a global level, research on the subject analyzed continues to evolve, so this study indicates new directions in research: (i) entropy analysis in the exchange of information content in financial markets based on entropy analysis, (ii) the implications of COVID-19 on the efficiency of the financial market, (iii) entropy in the mechanism of action for the development of the circular economy, (iv) a framework for evaluating corporate sustainability through multi-criteria decision-making, (v) the utility of entropy in estimating commercial risks, (vi) barriers to the implementation of blockchain technology in supply chains, and (vii) the study of complex phenomena in the interdisciplinary development of accounting as a technique for capturing financial records.

In short, business organizations as complex systems (dynamic, open, and non-linear) will always be unstable and in imbalance and will even be in conditions of uncertainty as they are far from equilibrium. For this reason, if it is established that organizations are complex systems in perfect balance, they should adapt to the environment in which they carry out their activity, which is practically impossible, since organizations never adapt to society. However, society adapts to organizations with slow changes, technological advances, and new trends in the markets.

In this way, the evolution and innovation in this field of research was identified from the configuration of the groups of authors, research institutions, countries/territories, and keywords, in relation to the intensity of the relationships that were developed in them. Hence, the results obtained are a complement to the knowledge on business information–entropy correlation in the accounting process of organizations and allow for the establishment of a link between science and business practice, to favor the decision-making process. In addition, the results of this study can provide supporting data for the development of future research and for decision-making to research institutions or financial organizations.

On the other hand, this analysis has a set of limitations, which conditioned the findings obtained, and they could be considered as a basis for future research work. Among the limitations are the use of the Scopus database to select the sample of scientific articles, the basic concepts chosen to search the aforementioned database, the temporal resumption, the statistical and mathematical techniques used, the software used for the construction and visualization of bibliometric networks, and the variables studied.

Finally, it is observed that research on the business information–entropy correlation in the accounting process of organizations is currently presenting an upward trend, according to the number of articles published, current lines of research, and future directions, which allows a growing trend in interest on the subject by the academic and scientific community worldwide to be deduced.

## Figures and Tables

**Table 1:** Main literature review to define the research object and the basic concepts.

Ref.	Year	Article Title	Author(s)	Journal	BC
[22]	2017	An approach to the evaluation of the quality of accounting information based on relative entropy in fuzzy linguistic environments	Li et al.	*Entropy*	E-A-I
[23]	2014	A hybrid multiple criteria decision-making model for investment decision-making	Hsu	*Journal of Business Economics and Management*	E-B
[24]	2012	Performance evaluation of coal–electricity supply chain based on IAHP–entropy evaluation model	Wei-Wei et al.	*Advances in Information Sciences and Service Sciences*	E-B-I
[1]	2009	An economic order quantity model for an imperfect production process with entropy cost	Jaber et al.	*International Journal of Production Economics*	E-A
[25]	2006	Differential entropy and dynamics of uncertainty	Garbaczewski	*Journal of Statistical Physics*	E-B
[26]	2005	On the convergence of the cross-entropy method	Margolin	*Annals of Operations Research*	E-B
[27]	2001	Updating and estimating a social accounting matrix using cross-entropy methods	Robinson et al.	*Economic Systems Research*	E-A
[28]	1997	An approach for measuring the degree of comparability of financial accounting information	Krisement	*European Accounting Review*	E-A-B-I
[29]	1993	Construct validity of an objective (entropy) categorical measure of diversification strategy	Hoskisson et al.	*Strategic Management Journal*	E-B
[30]	1982	A thermodynamic approach to economics	Bryant	*Energy Economics*	E-A-B

Ref.: reference; Year: year of publication; BC: basic concept; E: entropy; A: accounting; B: business; I: information.

**Table 2:** Evolution of the number of articles published per 5 years (1974–2020).

Period	Number of Articles	%	% Accumulated
2011–2020	654	66.73%	100.00%
2001–2010	197	20.10%	33.27%
1991–2000	78	7.96%	13.16%
1981–1990	35	3.57%	5.20%
1971–1980	16	1.63%	1.63%
Total	980	100.00%	

**Table 3:** Journal clusters (1974–2020).

Cluster			Journal	Weight		
Number	%	Color		Links	Total Link Strength	Citations
1	35.36%	Pink	*Econometrica*	231	5998	380
			*Journal of Econometrics*	181	5153	270
			*Journal of the American Statistical Association*	179	3124	162
			*Bell System Technical Journal*	242	2194	128
			*Journal of Finance*	134	2667	127
			*Entropy*	243	2194	114
2	27.62%	Green	*Journal of Cleaner Production*	79	6180	218
			*Information Sciences*	93	4070	175
			*Knowledge-Based Systems*	63	4549	150
			*Expert Systems with Applications*	111	3863	134
			*Science*	203	1798	111
			*IEEE Transactions on Fuzzy Systems*	70	2597	70
3	22.65%	Red	*European Journal of Operational Research*	210	5519	245
			*Expert Systems with Applications*	158	5168	233
			*International Journal of Production Research*	130	3568	158
			*Fuzzy Sets and Systems*	160	3180	152
			*International Journal of Production Economics*	139	2594	94
			*Tourism Management*	96	1215	88
4	14.36%	Yellow	*Management Science*	213	3167	141
			*Strategic Management Journal*	115	3149	140
			*Decision Support Systems*	136	2105	108
			*Academy of Management Journal*	88	1631	64
			*Research Policy*	76	775	58
			*Harvard Business Review*	116	899	49

%: percentage of journals that the cluster contains over the total; color: See Figure 3.

**Table 4:** Authors with more than 5 articles published (1974–2020).

Author	A	Research Institution	City (Country)	Keyword 1	Keyword 2	Keyword 3
Golan, A.	12	Santa Fe Institute	Santa Fe (USA)	Maximum entropy	Generalized maximum entropy	Censored data
		American University Department of Economics	Washington, D.C. (USA)			
Judge, G.	12	University of California	Berkeley (USA)	Information theoretic methods	Adaptive behavior	Empirical likelihood
Leydesdorff, L.	9	The Amsterdam School of Communications Research-ASCoR	Amsterdam (Netherlands)	Triple helix	Information theory	Knowledge-based systems
Soofi, E.S.	9	Lubar School of Business	Milwaukee (USA)	Kullback–Leibler information	Dirichlet process	Model fitting
Gossner, O.	6	London School of Economics and Political Science	London (UK)	Incomplete information	Relative entropy	Bayesian learning
		Institut Polytechnique de Paris	Palaiseau (France)			
Bailey, K.D.	5	University of California Department of Sociology	Los Angeles (USA)	Living systems	Social entropy theory	Behavioral research
Gzyl, H.	5	IESA, Center for Finance	Caracas (Venezuela)	Maximum entropy in the mean	Commerce	Costs
Maasoumi, E.	5	Emory University Department of Economics	Atlanta (USA)	Adaptive estimation	Computer simulation	Correlation methods
Miller, D.J.	5	Iowa State University Department of Economics	Ames (USA)	Maximum entropy	Adaptive behavior	Controlled stochastic process
Qian, Y.	5	Shanxi University	Taiyuan (China)	Rough set theory	Attribute reduction	Decision systems
Robinson, S.	5	Peterson Institute for International Economics	Washington, D.C. (USA)	Bayesian estimation	Computable general equilibrium (CGE)	Economic conditions
Tavana, M.	5	La Salle University Distinguished Chair of Business Analytics	Philadelphia (USA)	Decision support systems	Analytic network process	Business excellence
		Universität Paderborn Business Information Systems Department	Paderborn (Germany)			
Xu, Z.	5	Sichuan University, School of Business	Chengdu (China)	Decision-making	Aggregated information	Aleatory and epistemic uncertainties

A: number of articles; USA: United States; UK: United Kingdom.

**Table 5:** Author clusters (1974–2020).

Cluster			Author	Weight			
Number	%	Color		Links	Total Link Strength	Occurrences	Citations
1	59.43%	Pink	Wang L.	15	18	8	145
			Wang Y.	18	19	8	108
			Wang J.	21	21	6	67
			Li J.	20	20	5	201
			Qian Y.	12	16	5	169
2	20.90%	Green	Zhang S.	20	20	5	78
			Chen J.	13	13	4	32
			Dai J.	7	9	3	213
			Fujita H.	12	12	3	85
			Jiang H.	6	8	3	25
3	12.30%	Red	Li Y.	23	25	8	108
			Wang Z.	22	24	7	205
			Liu Y.	22	22	6	71
			Yang J.	1	1	4	25
			Li D.	7	8	2	85
4	4.92%	Yellow	Golan A.	4	6	12	380
			Judge G.	5	10	12	183
			Soofi E.S.	5	7	9	235
			Maasoumi E.	3	3	5	300
			Miller D.J.	1	1	5	86
5	2.46%	Purple	Wu J.	6	6	2	47
			Chen Y.-W.	5	5	1	28
			Liu X.-B.	5	5	1	28
			Qian X.-F.	5	5	1	28
			Yang J.-B.	5	5	1	28

%: percentage of journals that the cluster contains over the total; color: See Figure 4.

**Table 6:** Top 10 research institutions and main keywords (1974–2020).

Research Institution	City (Country)	A	Keyword 1	Keyword 2	Keyword 3
University of California Berkeley	Berkeley (USA)	16	Information theoretic methods	Adaptive behavior	Empirical likelihood
City University of Hong Kong	Kowloon (Hong Kong)	12	Decision-making	Fuzzy sets	Maximum entropy methods
Sichuan University	Sichuan (China)	12	Decision-making	Fuzzy sets	Decision theory
Ministry of Education China	Beijing (China)	11	Information entropy	Rough set	Artificial intelligence
Universiteit van Amsterdam	Noord-Holland (Netherlands)	11	Triple helix	Information theory	Knowledge-based systems
Universidad de Oviedo	Oviedo (Spain)	10	Empirical analysis	Decomposition analysis	Econometrics
University of Wisconsin-Milwaukee	Milwaukee (USA)	10	Kullback–Leibler information	Dirichlet process	Model fitting
London School of Economics and Political Science	London (UK)	9	Relative entropy	Blackwell ordering	Divergence measures
Chinese Academy of Sciences	Beijing (China)	9	Transfer entropy	Decision-making	Information entropy
Lubar School of Business	Milwaukee (USA)	9	Kullback–Leibler information	Dirichlet process	Model fitting

A: number of articles; USA: United States; UK: United Kingdom.

**Table 7:** Country/territory clusters (1974–2020).

Cluster			Country	Weight			
Number	%	Color		Links	Total Link Strength	Occurrences	Citations
1	38.71%	Pink	USA	40	137	281	8279
			China	23	80	190	5148
			India	16	27	89	945
2	29.03%	Green	Italy	13	22	42	649
			Iran	7	10	36	605
			Spain	19	40	32	439
3	19.35%	Red	France	19	38	33	632
			Switzerland	7	7	7	100
			Belgium	4	4	6	52
4	8.06%	Yellow	Canada	17	35	37	748
			Saudi Arabia	7	8	6	153
			Poland	3	3	5	30
5	4.84%	Purple	South Africa	5	7	4	25
			Kenya	2	2	1	2
			Uganda	2	2	1	2

%: percentage of journals that the cluster contains over the total; color: See Figure 5; USA: United States.

**Table 8:** Keyword clusters (1974–2020).

Cluster			Keyword	Weight		
Number	%	Color		Links	Total Link Strength	Occurrences
1	33.77%	Pink	Information theory (*)	139	238	86
			Thermodynamics	37	69	20
			Uncertainty	51	65	18
			Probability	48	69	17
			Maximum entropy analysis	39	54	16
			Supply chains	37	51	12
2	25.00%	Green	Maximum entropy (*)	96	151	56
			Mathematical models	86	172	26
			Optimization	64	86	17
			Probability distributions	61	111	17
			Computer simulation	67	99	14
			Economics	55	82	14
3	20.78%	Red	Information entropy (*)	71	107	44
			Data mining	62	95	24
			Rough set theory	44	98	18
			Learning systems	56	79	14
			Artificial intelligence	56	73	12
			Decision trees	37	56	11
4	17.86%	Yellow	Decision-making (*)	149	336	73
			Fuzzy sets	104	191	37
			Shannon entropy	43	52	27
			Efficiency	51	62	15
			Decision theory	39	57	12
			Sensitivity analysis	33	46	11
5	2.60%	Purple	Enthalpy (*)	9	26	6
			Controlled study	8	22	5
			Hydrophobicity	7	25	5
			Binding affinity	7	20	4
			Kinetics	7	17	4
			Quantitative analysis	22	22	4

%: percentage of keywords that the cluster contains of the total; color: See Figure 6; (*): keyword with the highest number of occurrences within each cluster, which gives the group its name.

## Data Availability

The data were obtained from Elsevier’s Scopus database (https://www.scopus.com/), accessed on 1 October 2021.
